# Rescue epilepsy medication and training: A comparison between midazolam use, guidelines, clinical practice, and possibilities in the UK and Norway

**DOI:** 10.1002/epi4.70145

**Published:** 2025-10-06

**Authors:** Audrey McBride, Oliver Johannes Henning, Cecilie Johannessen Landmark, Lance Watkins, Samuel Tromans, Paul Basset, Paraskevi Triantafyllopoulou, Rohit Shankar

**Affiliations:** ^1^ Exeter Medical School University of Exeter Truro UK; ^2^ National Center for Epilepsy Oslo University Hospital Oslo Norway; ^3^ Peninsula School of Medicine University of Plymouth Truro UK; ^4^ Swansea Bay University Health Board Neath Port Talbot UK; ^5^ University of Leicester Leicester UK; ^6^ Adult Learning Disability Service, Leicestershire Partnership NHS Trust Leicester UK; ^7^ Statsconsultancy Ltd Amersham UK; ^8^ Tizard Centre, University of Kent Canterbury UK; ^9^ Cornwall Intellectual Disability Equitable Research (CIDER) Cornwall Partnership NHS Foundation Trust Truro UK

**Keywords:** benzodiazepine, epilepsy mortality, epilepsy risk, prolonged seizures, rescue therapy

## Abstract

**Objectives:**

Status epilepticus (SE) is a prolonged epileptic seizure carrying significant risk of long‐term disability and death. Rescue therapies are prescribed for prehospital administration to terminate SE. This study compared prescribing practices of rescue therapies of midazolam in the UK and Norway.

**Methods:**

A cross‐sectional, online, 21‐item Likert‐style survey was administered to epilepsy professionals in the UK circulated via ILAE/ESNA and in Norway via ILAE/Epilepsinet using a non‐discriminatory exponential snowballing technique leading to non‐probability sampling. Data were collected anonymously and analyzed using descriptive statistics, Mann–Whitney, Chi‐square, and Fisher's exact tests. Significance was accepted at *p* < 0.05.

**Results:**

All 86 UK and 53 Norway respondents identified buccal midazolam as the first‐line rescue medication choice in the community for prolonged and/or generalized tonic–clonic seizures. Norwegian respondents had significantly more experience in epilepsy‐related work (*p* = 0.002), were more likely to have a larger caseload on buccal midazolam (*p* < 0.001), prescribed higher midazolam doses (*p* < 0.001), and provided training yearly (*p* < 0.001). UK respondents were more likely to delegate rescue medication prescribing to primary care (*p* = 0.006) and reviewed emergency management plans more frequently (*p* = 0.006). There was an inter‐country difference in the period of midazolam non‐use that respondents required before withdrawing from treatment plans (*p* < 0.001). Concern about inappropriate use of buccal midazolam was similarly high in both countries.

**Significance:**

This study compared epilepsy professionals in two neighboring high‐income countries. Findings suggest an urgent need for international guidelines to recommend best practices on prescribing doses and withdrawal of buccal midazolam. The potential abuse of buccal midazolam by patients and carers warrants further investigation.

**Plain Language Summary:**

This study investigated doctors' and nurses' views on the use of epilepsy rescue therapies, particularly midazolam. These are medications that can stop dangerously long epileptic seizures from progressing to the point of serious injury or death. The study was carried out in both the UK and Norway to establish differences between the countries. The key findings were that Norwegian doctors prescribed rescue medications at a higher dose, that UK clinicians reviewed and deprescribed medications faster after patients stopped having prolonged seizures, and that clinicians in both countries were concerned about medications being misused or abused by patients and carers.

**Key points**

- People with epilepsy are at risk of status epilepticus (SE), a prolonged seizure that can lead to serious neuronal damage and be fatal.
- SE is treated with therapies including buccal midazolam, which is the international gold standard, but inter‐country differences in its use exist.
- Norwegian respondents prescribed buccal midazolam at higher doses and amidst a greater range of comorbidities than UK respondents.
- UK respondents reviewed emergency management plans more frequently and withdrew midazolam more quickly than Norwegian respondents.
- Both countries' respondents were highly concerned about the inappropriate use/abuse of midazolam, which requires further investigation.

## 1 INTRODUCTION

With an estimated age‐standardized prevalence of 658/100 000 people worldwide, epilepsy is one of the most prevalent neurological conditions globally and has among the most significant disease burdens, as quantified by global disability‐adjusted life years.^1^, ^2^

People with epilepsy have a two to three times higher risk of premature death than the general population.^3^ Approximately 10% of epilepsy‐related deaths are due to status epilepticus (SE), an epileptic seizure of extended duration.^3^ This varies by seizure type but is 5 min and over for tonic–clonic seizures.^4^ If SE persists to a second time point (e.g., 30 min for a tonic–clonic seizure), the seizure may cause irreparable neuronal injury, neuronal death, and fatal functional impairments.^4^ In addition, there is developing evidence of SE being associated with the other major cause of death in people with epilepsy, that is, Sudden Unexpected Death in Epilepsy.^5^ All people with epilepsy are at risk of prolonged seizures, SE, and its consequences.

Emergency pharmacological treatments, also called *rescue therapies*, can be administered to disrupt seizures and avoid the progression of SE.^6^ These are lifesaving medications that can be prescribed for emergency use outside of hospital environments by suitably trained people as part of a personalized emergency management plan.

Buccal midazolam is the best evidenced rescue therapy for tonic–clonic seizures, both in its effectiveness and straightforward, dignified administration compared to other routes including rectal.^6^, ^7^, ^8^ In high‐income countries (HICs), particularly in Western Europe, midazolam has been widely adopted. Midazolam is given as an oromucosal solution, 2.5, 5, 7.5 or 10 mg in a prefilled syringe from 0.5 to 2 mL, where 10 mg is the most commonly used dose in adults.^9^ The given indication is prolonged, acute, convulsive seizures from 3 months of age onwards.^9^, ^10^ The absorption is rapid and the bioavailability is about 75% in adults and up to 87% in children, and the initial half‐life is about 30 min, allowing a rapid onset and termination of the effect.^9^

In the UK, the National Institute of Health and Care Excellence (NICE) provides guidelines on rescue therapy indications and prescribing, while the Epilepsy Specialist Nurses Association (ESNA), Royal College of Psychiatrists, and International League Against Epilepsy (ILAE) have co‐produced guidelines on rescue therapy administration and administrator training.^8^, ^10^ These standards are considered best practice globally, particularly in HICs. However, internationally, there are major gaps in evidence and clinical guidelines concerning first‐line rescue therapies for different seizure types, maximum buccal midazolam dosage, cautions and contraindications, abuse potential, and how and when to withdraw buccal midazolam from a treatment plan.^11^

### 1.1 Comparing rescue therapy guidance between two neighboring HICs, that is, UK and Norway

UK and Norway, though having differences in population and health systems, are comparable across diverse socio‐demographic and health delivery outcomes.^12^ In the United Kingdom (UK) (population: 67.3 million), there are 1200 epilepsy‐related deaths a year, while in Norway (population: 5.4 million), it is estimated there are around 130 epilepsy‐related deaths each year.^13^, ^14^ Recent studies of comparison between the two countries have shown similarities and differences in other areas of epilepsy risk management.^15^

The first‐line prehospital rescue therapy in the UK is 10 mg of buccal midazolam, with a further 10 mg dose 5–10 min later, if SE persists.^6^ Individuals requiring rescue therapies have emergency seizure management plans formulated by specialist nurses or doctors, with either type of clinician prescribing rescue therapies for prehospital use.^6^, ^10^ These plans should be available in schools and working environments.

In Norway, national guidelines recommend the use of benzodiazepines, either buccal, IM, or IV midazolam (10 mg) or diazepam (0.25 mg/kg) either IV or rectal, as a prehospital rescue therapy. Buccal midazolam is recommended for people with known epilepsy as first‐line pharmacological rescue therapies in the prehospital setting.^16^ Usually, treating physicians prescribe a rescue medicine to patients considered at risk for SE. Epilepsy nurses follow up with people with epilepsy in an outpatient setting, but they cannot prescribe any rescue medications.

The REMIT (Rescue Epilepsy Medication and Training) survey was administered firstly to UK and secondly to Norway‐based epilepsy professionals. This was done to understand and compare the reported prescribing and clinical practices strategies of the UK and Norway.

## 2 METHODS

Due to slight variation in content and administration, the methodology of each country's survey is reported separately, in accordance with the Checklist for Reporting Results of Internet E‐Surveys (CHERRIES) checklists (Tables S1 and S2). In the UK, the survey was open between 13/10/2023 and 29/12/2023. In Norway, the survey was open between 01/06/2024 and 05/09/2024.

### 2.1 Development

The survey was developed iteratively, using informal interviews of epilepsy specialists and key documents pertaining to UK‐based rescue therapy regulation.^5^ The initials of collaborators are given in parentheses. A preliminary draft was adjusted across five review cycles. It was then hosted online and completed by the Epilepsy Specialist Nurses Association (ESNA) executives to evaluate usability and content before finalization in September 2023. The UK survey tool was translated to Norwegian by two experienced Norwegian epileptologists. A copy of the survey can be found in Data S1.

### 2.2 Distribution, administration, design

In the UK, the survey was disseminated using an exponential, non‐discriminative snowball methodology, with recipients encouraged to share it further in order to capture a non‐probability sample. It was distributed via newsletter emails to ESNA and British ILAE members.

In Norway, the survey was distributed through collaboration with key stakeholder organizations, including the Norwegian chapter of the ILAE, the national network for epilepsy nurses, and the national interdisciplinary epilepsy network (Epilepsinet). The survey was distributed as an electronic form (Questback), and data were collected anonymously.

### 2.3 Ethics and governance

In the UK, the survey was reviewed by professional bodies (ESNA and ILAE) before being administered to an exclusively to their members who are a professional audience. Informed consent was gained on the survey's introductory page. In Norway, the survey was reviewed by the Regional Ethics Committee and the local body for evaluation of privacy. Both entities approved the study with reference numbers 723044 and 24/01100, respectively.

### 2.4 Statistical analysis

Analysis used the Stata software package (version 15.1). The analysis compared questionnaire responses between UK and Norwegian respondents. Categorical variables with no ordering to the categories were compared between groups using the Chi‐square test or Fisher's exact test. Categorical variables where there was a natural ordering to the categories were compared between staff groups using the Mann–Whitney test. Only like‐for‐like survey items were included in the comparative analysis. The level of significance was set at *p* < 0.05.

## 3 RESULTS

### 3.1 Demographics

Table 1 summarizes the key characteristics of the study respondents from the two countries. More specific details on participant demographics can be found in Tables S3 and S4. In total, there were 86 respondents from the UK and 53 from Norway. Just over a half (57%; *n* = 30) of Norwegian respondents were physicians, as compared to about a quarter (24%; *n* = 21) of UK respondents (*p* < 0.001). The remaining respondents in both countries were epilepsy nurses.

**TABLE 1:** Characteristics of study respondents of the UK and Norway.

Variable	Category	UK		Norway		*p*‐Value
		*N*	Number (%)	*N*	Number (%)	
Job category	Physician	86	21 (24%)	53	30 (57%)	**<0.001**
	Nurse		64 (74%)		21 (40%)	
	Other		1 (1%)		2 (4%)	
Experience in epilepsy role	0–3 years	86	16 (19%)	53	1 (2%)	**0.002**
	3–5 years		17 (20%)		7 (13%)	
	5–10 years		11 (13%)		7 (13%)	
	10+ years		42 (49%)		38 (72%)	
Epilepsy‐specific work	<25%	86	7 (8%)	53	8 (15%)	0.53
	25%–50%		15 (17%)		6 (11%)	
	50%–75%		11 (13%)		9 (17%)	
	>75%		53 (62%)		30 (57%)	

*Note*: Bold values significance taken at *p* <0.05.

### 3.2 Training families and carers in epilepsy risk and administration of rescue therapy medication

There were no significant inter‐country differences in the proportion of respondents who provided epilepsy‐related training or in the method of training delivery to family members and care providers of people with epilepsy on risk and use of rescue therapies (Table 2). However, almost two‐thirds of Norwegian respondents (64%; *n* = 27) provided training more than once a year compared to only 21% (*n* = 14) of UK respondents (*p* < 0.001). UK respondents were more likely to delegate rescue medication prescribing from specialist to primary care than Norwegian respondents (*p* = 0.006).

**TABLE 2:** Training in epilepsy risk and prescribing rescue therapies for community UK versus Norway (all respondents).

Variable	Category	UK		Norway		*p*‐Value
		*N*	Number (%)	*N*	Number (%)	
Provide training in epilepsy	No	86	15 (17%)	52	9 (17%)	0.98
	Yes		71 (83%)		43 (83%)	
Mode of training delivery^a^	F‐t‐f + Virtual	71	30 (42%)	43	20 (47%)	0.66
	Face‐to‐face		41 (58%)		23 (53%)	
Frequency of training^a^	>Annually	67	14 (21%)	42	27 (64%)	**<0.001**
	Annually		21 (31%)		6 (14%)	
	Every 2 years		15 (22%)		0 (0%)	
	<2 years		4 (6%)		5 (12%)	
	As required		13 (19%)		4 (10%)	
Prescribe rescue therapy	No	70	40 (57%)	53	24 (45%)	0.19
	Yes		30 (43%)		29 (55%)	
Request GP to prescribe^b^	No	30	4 (13%)	28	13 (46%)	**0.006**
	Yes		26 (87%)		15 (54%)	

*Note*: Bold values significance taken at *p* <0.05.

^a^ Figures for respondents providing training only.

^b^ Figures for those who prescribe rescue therapy only.

### 3.3 Use of emergency management plans

Table S5 provides details of the initiation and review of emergency management plans UK versus Norway for all respondents. There were no inter‐country differences in the reasons for implementing an emergency management plan. Notably, respondents indicated that emergency management plans were reviewed more frequently in the UK, with 87% of respondents indicating that they would review the plan annually or more frequently, compared to Norway (55%) (*p* = 0.006).

### 3.4 Prescribing of buccal midazolam for community use

**FIGURE 1:**
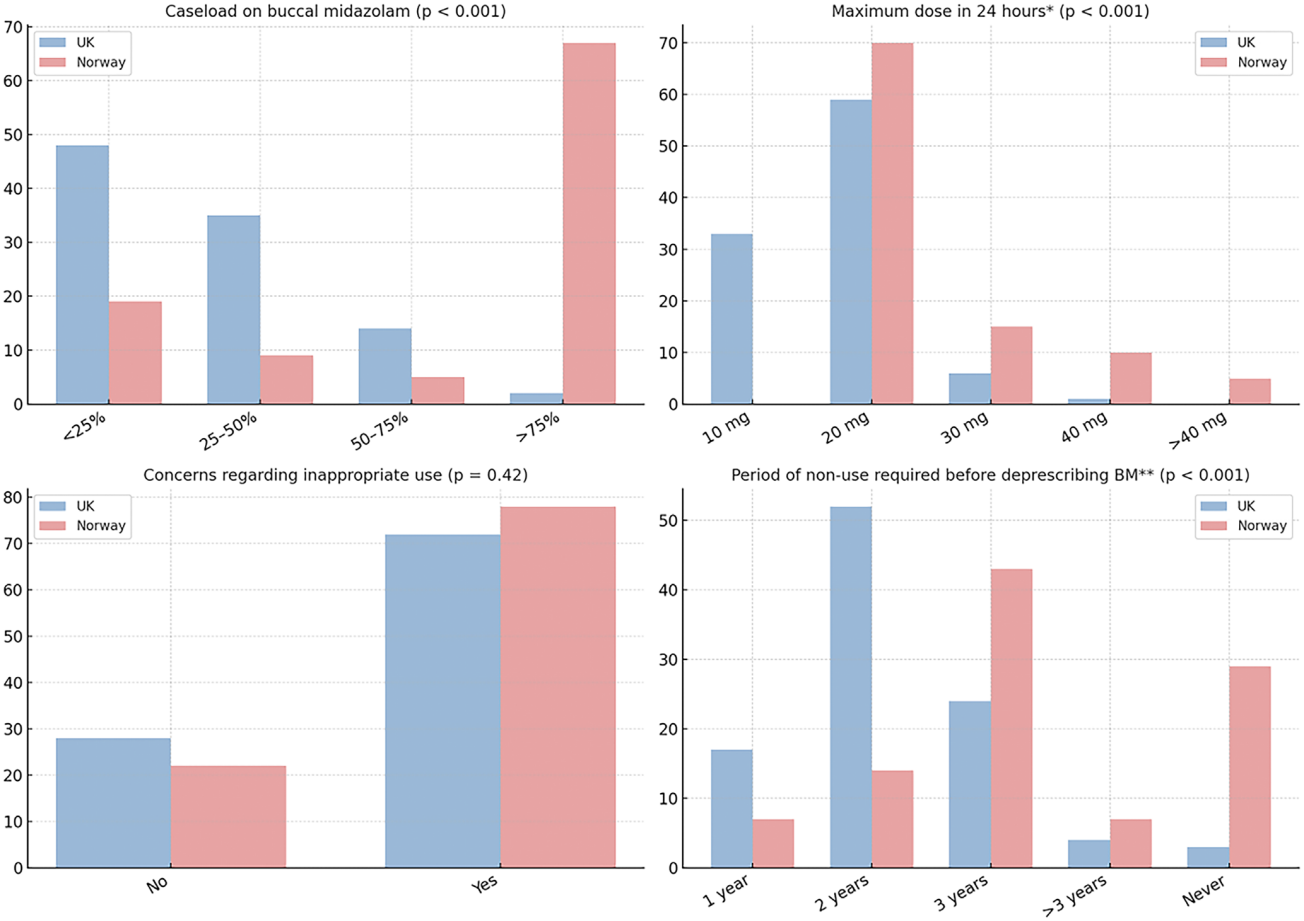
Buccal midazolam items, UK vs Norway (all respondents).

Figure 1 shows the comparison between UK and Norway respondents across all buccal midazolam items. The maximum dose reportedly prescribed by respondents was significantly higher in Norway (*p* < 0.001). Approximately a third of respondents (30%, *n* = 12) indicated that they would give ≥30 mg buccal midazolam within 24 h, and none would prescribe less than 20 mg/24 h. In the UK, only 7% (*n* = 6) of respondents would prescribe more than 30 mg in 24 h, and 33% (*n* = 26) would prescribe a maximum of 10 mg in 24 h.

There was a significant inter‐country difference in the period of buccal midazolam non‐use that respondents required before withdrawing buccal midazolam from treatment plans (*p* < 0.001). In the UK, 69% (*n* = 49) of respondents indicated that they would withdraw buccal midazolam after up to 2 years of non‐use, compared to only 21% (*n* = 9) of Norwegian respondents. Most Norwegian respondents (79%, *n* = 33) indicated that they required a period of non‐use of ≥3 years, with just under a third (29%, *n* = 12) of respondents reporting that they would never withdraw buccal midazolam from an emergency plan, regardless of the period of non‐use.

Concern about inappropriate use of buccal midazolam was similarly high in both countries and expressed by 72% (*n* = 58) and 78% (*n* = 39) of UK and Norwegian respondents, respectively.

Figure S1 compares UK and Norway physicians with regard to their buccal midazolam items. Although the sample was small, significant differences emerged. Just over a quarter of UK physicians (26%, *n* = 5) prescribed a maximum dose of 10 mg buccal midazolam/24 h, compared to 0 Norwegian physicians, while only 1 UK physician prescribed ≥40 mg buccal midazolam/24 h, compared to seven (14%) of Norwegian physicians (*p* = 0.008). The majority of the physicians in the UK (63%, *n* = 12) and Norway (70%, *n* = 21) chose 20 mg/24 h as their custom prescribing dose.

Significant differences in time to withdrawal after non‐use of buccal midazolam were seen between UK and Norway physicians (*p* = 0.009) with 50% (*n* = 7) of UK physicians customarily withdrawing midazolam in two or fewer years of use compared to 17% (*n* = 5) from Norway.

### 3.5 Prescribing in specific scenarios

**FIGURE 2:**
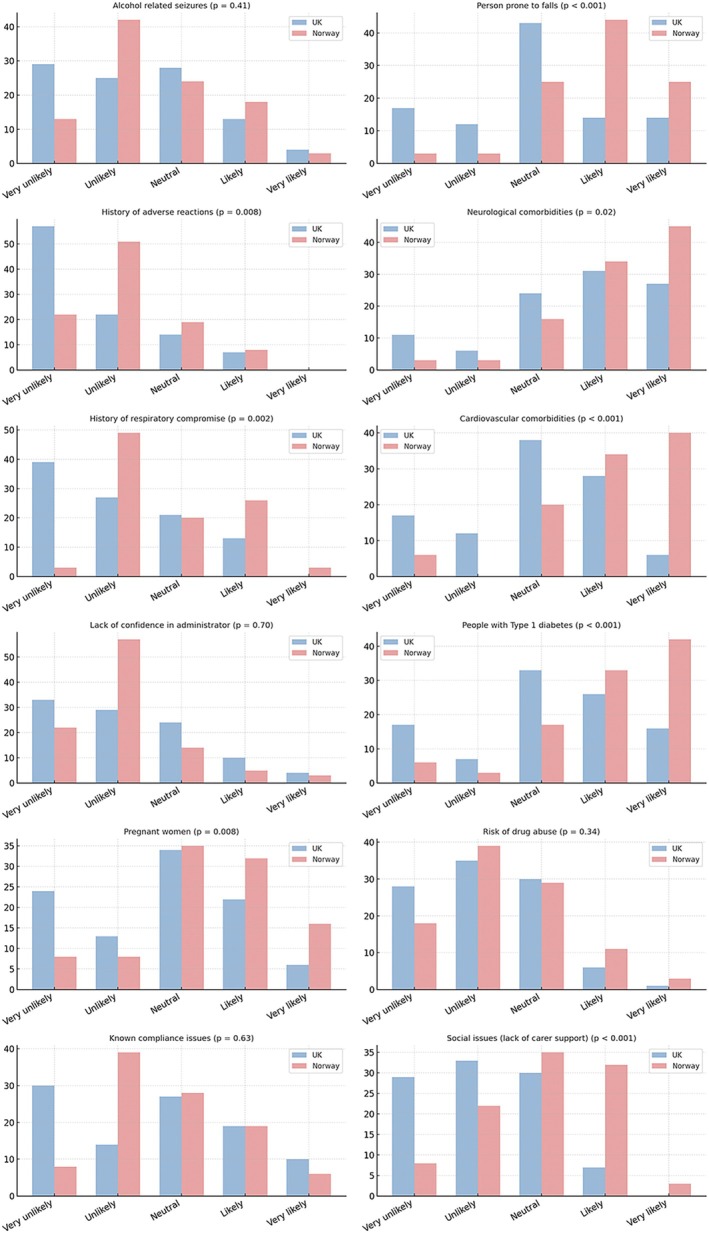
Likelihood of prescribing buccal midazolam in different scenario, UK vs Norway (all respondents).

Figure 2 shows the distribution of responses between UK‐ and Norway‐based respondents in relation to prescribing in specific scenarios, and Figure S2 shows the responses among physician respondents only.

Respondents from both countries were overall unlikely (i.e., >50% respondents of each country) to prescribe buccal midazolam for people with a history of alcohol‐related seizures (UK 54%, Norway 55%), adverse reactions to buccal midazolam (UK 79%, Norway 73%), history of respiratory compromise (UK 66%, Norway 52%), risk of drug abuse (UK 63%, Norway 57%), or where there was a lack of confidence in the patient's administrator (e.g., family member) (UK 62%, Norway 79%).

There were no inter‐country differences in attitudes for prescribing buccal midazolam to people with epilepsy (or their carers) with known compliance concerns. In the UK group, 44% and in the Norway cohort, 47% felt they were unlikely to prescribe.

Significant inter‐country differences between respondents emerged from almost all items inquiring about the likelihood of prescribing buccal midazolam for patients with different comorbidities or other contextual factors. Norwegian respondents were significantly more likely to prescribe buccal midazolam for patients with a history of neurological comorbidities (*p* = 0.02), pregnant women (*p* = 0.008), cardiovascular comorbidities (*p* < 0.001), Type 1 diabetes mellitus (*p* < 0.001), patients prone to falls (*p* < 0.001), and those with a lack of carer support (*p* < 0.001).

When comparing physicians alone (Figure S2) on prescribing to different comorbidities, the only significant inter‐country difference was Norwegian physicians being more likely (*p* = 0.04) than their UK peers to prescribe buccal midazolam for patients with a lack of carer support.

## 4 DISCUSSION

### 4.1 Demographic differences

Epilepsy healthcare services in Norway and the UK are structured differently, and this is reflected in respondents' clinical backgrounds. There were proportionately far more physician respondents in Norway than the UK, and far more epilepsy specialist nurses in the UK than in Norway. This makes the two subgroups heterogeneous with respect to professional background, but representative of the relative influence of each clinician group on each country's epilepsy services.^8^

### 4.2 Use of buccal midazolam and emergency plans

Midazolam, while appropriate to use in the community, also has significant potential for benzodiazepine habituation, dependence, and abuse.^17^, ^18^, ^19^ A cross‐sectional analysis conducted by the National Survey on Drug Use and Health adult data showed that the misuse of benzodiazepines accounted for 17.2% of the overall use of benzodiazepines.^20^ In our study, UK and Norwegian clinicians both shared a high level of concern about the abuse and misuse of buccal midazolam. Previous research qualitatively analyzed UK clinicians' concerns and highlighted buccal midazolam's abuse potential.^21^ However, there has generally been very limited inquiry into this issue, particularly around safety standards to prevent misuse.

Norwegian clinicians preferred a far longer period of non‐use of buccal midazolam before deprescribing it, and this difference persisted in a physician‐only comparison between Norway and the UK. This could be accounted for by the aforementioned high proportion of neurologists among Norwegian respondents, who likely manage patients at a higher risk of seizure recurrence or with complex issues including nonadherence to antiseizure medication.^22^ In the UK, epilepsy specialist nurses would oversee those who have complex but stable epilepsy, possibly giving more opportunity to deprescribe medication sooner.

Overall, emergency plans were reviewed more frequently in the UK than in Norway, which could also could drive the earlier deprescribing in the UK. Another factor is the contrasting geography of the UK and Norway. Norwegian physicians' practice is influenced by the need to accommodate rurally located patients, who may require helicopter and ambulance boats to reach the hospital, with associated long wait times and the need for caution even in low‐risk scenarios, which is also seen in other conditions.^23^, ^24^, ^25^ It is recognized that antiseizure medication discontinuation is also delayed in Norway, and that clinicians are reluctant to discuss discontinuation.^26^ While deprescribing should always be guided by individual need, clinicians may benefit from more research establishing reliable criteria for deprescribing, and services may benefit from an economic analysis of long‐term prescribing, despite buccal midazolam's competitive cost‐effectiveness.^27^ Further, the lack of clear international guidelines on when to withdraw midazolam adds to the risk of misuse and abuse at patient and carer levels.

### 4.3 Prescribing practices

Norwegian clinicians indicated a preference for a higher maximum dose of buccal midazolam over 24 h compared to UK clinicians, and this persisted in physician‐only analyses. The prescription patterns and individual indications for antiseizure medications in Norway and the UK have been well studied.^6^, ^28^, ^29^, ^30^ Future research should investigate prehospital therapies, including optimal dosing and indications. International comparisons of practice could help establish the rationale for, and frequency of, prescribing doses outside of guidelines.

Norwegian clinicians were overall more likely to prescribe buccal midazolam to patients with multiple comorbidities or social challenges; however, this difference was driven by UK nurse respondents and did not persist in a physician‐only analysis, where physicians from both countries were equally likely to prescribe buccal midazolam in patients with multimorbidity. This finding further underlines the need to review UK guidelines and strengthen the evidence base for prescribing in patients in various health states, including those who are pregnant, have type 1 diabetes, or cardiovascular comorbidities, to ensure appropriate and equitable buccal midazolam prescribing in the UK.^21^, ^31^

### 4.4 Strengths and limitations

#### 4.4.1 Strengths

This is the first research paper to perform an international comparison of prehospital, pharmacological seizure rescue therapies, with a focus on buccal midazolam. It provides insights into clinical practice in an area without a clear evidence base or published guideline, which is particularly relevant to deprescribing and prescribing in patients with multimorbidity. The survey was translated and adapted by expert neurologists for deployment in Norway, optimizing usability and results. The diversity of professional background among respondents realistically represents the clinician body of each country's epilepsy service, including their clinical decisions and influences. It highlights a hitherto unrecognized concern of potential misuse and abuse of midazolam.

#### 4.4.2 Limitations

Any survey cannot demonstrate causation, only association. Although this survey was developed by experts in the field, there was no pilot testing performed prior to dissemination that may have helped check reliability based upon respondents' feedback. The survey tool did not include the health care setting of the participants, which could have helped bring insight into access to neurological care, resources, and the rural–urban divide.

The UK respondent sample is professionally more heterogeneous than Norway, and thus this study does not fully compare professionals with shared backgrounds, for example, neurologist with neurologist. The survey also compares very differently structured epilepsy services, as Norway has no nurse prescribing. However, it is a realistic sample of epilepsy services in two developed countries as delivered at the coalface. Furthermore, the translation of the survey from English to Norwegian may have resulted in some changed meaning, despite its best accuracy. This paper does not comparatively explore concerns about inappropriate use, which should be expanded upon in future work.

Notably, these differences are driven by within‐country differences in prescribing in comorbidity between physicians and nurses in the UK, which have been highlighted in previous research.^21^

## 5 CONCLUSIONS

This study compares two professionally distinct cohorts from differently structured epilepsy services across the UK and Norway. The most significant differences emerged in clinicians' maximal dose of buccal midazolam prescribed (higher in Norway), the period of non‐use before deprescribing (longer in Norway), and the likelihood of prescribing midazolam in multimorbidity (more likely in Norway). Both countries prescribed midazolam for similar indications. Both had very high concerns about inappropriate use and abuse of buccal midazolam. There is an urgent need for international standards in prescribing and deprescribing midazolam, as well as further research into the extent of misuse and abuse potential of midazolam by patients and carers.

## AUTHOR CONTRIBUTIONS

All authors satisfy the ICMJE guidance by substantially contributing to the design, analysis and interpretation of the work, drafting of the manuscript, final approval of the manuscript and all agree to be accountable for all aspects of the work in ensuring that questions related to the accuracy or integrity of any part of the work is appropriately investigated and resolved. AMB – conceptualization, project administration, validation, visualization, writing – original draft. OJH – conceptualization, data curation, investigation, project administration, resources, validation, visualization, and writing – review and editing. CJL – conceptualization, investigation, validation, visualization, and writing – review and editing. LW – data curation, investigation, validation, visualization, and writing – review and editing. ST – data curation, investigation, project administration, validation, visualization, and writing – review and editing. PB – formal analysis, investigation, methodology, resources, software, supervision, validation, visualization, and writing – review and editing. PT – formal analysis, investigation, methodology, resources, software, supervision, validation, visualization, and writing – review and editing. RS – conceptualization, formal analysis, investigation, methodology, project administration, resources, supervision, validation, visualization, writing – original draft, and writing – review and editing.

## FUNDING INFORMATION

None.

## CONFLICT OF INTEREST STATEMENT

There is no direct disclosure or conflict of interest for any author for this submitted body of work. OJH has received speakers' honoraria from Eisai, Roche, Jazz, and UCB pharma outside this work. CJL has received speaker/expert group honoraria from Angelini, Eisai, Jazz, and UCB pharma outside this work. LW has received speakers' honoraria from UCB and Veriton pharma outside this work. RS developed the non‐commercial and free‐to‐use SUDEP and Seizure Safety Checklist and the EpSMon app to reduce the risk of SUDEP and enhance seizure safety. RS is the chief investigator of the NIHR‐adopted national Ep‐ID register. The register is supported and monitored by the National Institute of Health Research UK. The funding for each molecule examined by the register is via an investigator‐initiated support grant from each of the molecule's parent company. The funding is to RS's NHS institution and goes toward the salary of the research coordinator and the institution's project oversight costs. The contributing companies to date include Eisai, UCB, Bial, Jazz pharma (previously GW pharma), and Angelini. This work sits outside the submitted work. In addition to the above, RS has received institutional research, travel support, and/or honoraria for talks and expert advisory boards from LivaNova, UCB, Eisai, Neuraxpharm, Veriton pharma, Bial, Angelini, UnEEG, and Jazz/GW pharma outside the submitted work. He holds or has held competitive grants from various national grant bodies including Innovate, Economic and Social Research Council (ESRC), Engineering and Physical Sciences Research Council (ESPRC), National Institute of Health Research (NIHR), NHS Small Business Research Initiative (SBRI), and other funding bodies including charities—all outside this work. No other author has any declared conflict of interest related to this paper.

## ETHICS STATEMENT

We confirm that we have read the journal's position on ethical approval and affirm that this report is consistent with those guidelines. Multi‐country approvals have been taken or where not needed explained as such and have outlined in methods.

## Supporting information


Data S1.

Table S1.

Table S2.

Table S3.

Table S4.

Table S5.

Figure S1.

Figure S2.

## Data Availability

The data that support the findings of this study are available from the corresponding author upon reasonable request.

## References

[1] GBD Epilepsy Collaborators . Global, regional, and national burden of epilepsy, 1990–2021: a systematic analysis for the global burden of disease study 2021. Lancet Public Health. 2025;10(3):e203–e227. 10.1016/S2468-2667(24)00302-5 40015291 PMC11876103

[2] GBD 2021 Nervous System Disorders Collaborators . Global, regional, and national burden of disorders affecting the nervous system, 1990–2021: a systematic analysis for the global burden of disease study 2021. Lancet Neurol. 2024;23(4):344–381. 10.1016/S1474-4422(24)00038-3 38493795 PMC10949203

[3] Trinka E , Rainer LJ , Granbichler CA , Zimmermann G , Leitinger M . Mortality, and life expectancy in epilepsy and status epilepticus‐current trends and future aspects. Front Epidemiol. 2023;3:1081757. 10.3389/fepid.2023.1081757 38455899 PMC10910932

[4] Trinka E , Cock H , Hesdorffer D , Rossetti AO , Scheffer IE , Shinnar S , et al. A definition and classification of status epilepticus—report of the ILAE task force on classification of status epilepticus. Epilepsia. 2015;56(10):1515–1523. 10.1111/epi.13121 26336950

[5] Puras Z , Richardson S , Vincent Watkins L , Shankar R . Status epilepticus a risk factor for sudden unexpected death in epilepsy (SUDEP): a scoping review and narrative synthesis. Epilepsy Behav. 2024;160:110085. 10.1016/j.yebeh.2024.110085 39388974

[6] National Institute for Health and Care Excellence . Epilepsies in children, young people and adults: diagnosis and management [NG217]. 2022. 36395299

[7] Shankar R , Goodwin M , Toland J , Boyle A , Grant A , Pearson J , et al. Oro‐mucosal midazolam maleate: use and effectiveness in adults with epilepsy in the UK. Epilepsy Behav. 2021;123:108242. 10.1016/j.yebeh.2021.108242 34371288

[8] Tittensor P , Tittensor S , Chisanga E , Bagary M , Jory C , Shankar R . UK framework for basic epilepsy training and oromucosal midazolam administration. Epilepsy Behav. 2021;122:108180. 10.1016/j.yebeh.2021.108180 34252835

[9] European Medicines Agency . Buccolam product characteristics. 2016 May [cited 2025 Mar 23]. Available from: https://www.ema.europa.eu/en/documents/product‐information/buccolam‐epar‐product‐information_en.pdf

[10] Best practice guidelines for training professional carers in the administration of Buccal (Oromucosal) Midazolam for the treatment of prolonged and/or clusters of epileptic seizures in the community. Available from: https://esna‐online.org/wp‐content/uploads/2024/01/Midazolam‐interim‐guidelines‐MI_12454014_21.12.23_V_3.pdf (Accessed 30.03.2025)

[11] Shankar R , Jory C , Ashton J , McLean B , Walker M . Epilepsy emergency rescue training. BMJ Qual Improv Rep. 2015;4(1):u208167.w3566. 10.1136/bmjquality.u208167.w3566 PMC4645852 26734339

[12] Public Health England . The Burden of Disease in England compared with 22 peer countries a report for NHS England. Available from: https://assets.publishing.service.gov.uk/media/5e1735f2ed915d3b0b00c7cc/GBD_NHS_England_report.pdf

[13] Shankar R , Newman C , Gales A , McLean BN , Hanna J , Ashby S , et al. Has the time come to stratify and score SUDEP risk to inform PWE of their changes in safety? Front Neurol. 2018;9:281. 10.3389/fneur.2018.00281 29755403 PMC5934492

[14] Aurlien D , Larsen JP , Gjerstad L , Taubøll E . Increased risk of sudden unexpected death in epilepsy in females using lamotrigine: a nested, case‐control study. Epilepsia. 2012;53:258–266. 10.1111/j.1528-1167.2011.03334.x 22126371

[15] Watkins L , Henning O , Bassett P , Ashby S , Tromans S , Shankar R . Epilepsy professionals' views on sudden unexpected death in epilepsy counselling: a tale of two countries. Eur J Neurol. 2024;31(9):e16375. 10.1111/ene.16375 38837829 PMC11295158

[16] Johannessen Landmark C , Eyal S , Burns ML , Franco V , Johannessen SI . Pharmacological aspects of antiseizure medications: from basic mechanisms to clinical considerations of drug interactions and use of therapeutic drug monitoring. Epileptic Disord. 2023;25(4):454–471. 10.1002/epd2.20069 37259844

[17] https://www.cureepilepsy.org/understanding‐epilepsy/treatments/epilepsy‐medications/midazolam/#:~:text=The%20use%20of%20midazolam%20more,sleeplessness%2C%20nervousness%2C%20and%20seizures.

[18] Controlled drugs and drug dependence | Medicines guidance | BNF content published by NICE [Internet]. [cited 2025 Mar 14]. Available from: https://bnf.nice.org.uk/medicines‐guidance/controlled‐drugs‐and‐drug‐dependence/.

[19] Kroenke K , Hirschtritt ME . Walking the benzodiazepine high wire. Psychiatr Serv. 2022;74(1):73–75. 10.1176/appi.ps.202100671 36321316

[20] Maust DT , Lin LA , Blow FC . Benzodiazepine use and misuse among adults in the United States. Psychiatr Serv. 2019;70(2):97–106. 10.1176/appi.ps.201800321 30554562 PMC6358464

[21] McBride A , Watkins L , Tromans S , Triantafyllopoulou P , Basset P , Tittensor P , et al. The current clinical practice and experiences in buccal midazolam prescribing in community for status epilepticus termination in the United Kingdom: the rescue epilepsy medication and training (REMIT) study. Seizure. 2025;125:62–72. 39809115 10.1016/j.seizure.2024.12.022

[22] Henning O , Lossius MI , Lima M , Mevåg M , Villagran A , Nakken KO , et al. Refractory epilepsy and nonadherence to drug treatment. Epilepsia Open. 2019;4(4):618–623. 10.1002/epi4.12367 31819918 PMC6885656

[23] Rørtveit S , Meland E , Hunskaar S . Changes of triage by GPs during the course of prehospital emergency situations in a Norwegian rural community. Scand J Trauma Resusc Emerg Med. 2013;21:89. 10.1186/1757-7241-21-89 24354953 PMC3878323

[24] Kjærvoll HK , Andersson LJ , Bakkelund KEN , Harring AKV , Tjelmeland IBM . Description of the prehospital emergency healthcare system in Norway. Resusc Plus. 2023;17:100509. 10.1016/j.resplu.2023.100509 38076383 PMC10701116

[25] Hustad IA , Horn M , Rehn M , Taubøll E , Hov MR . Prehospital seizure management protocols need standardized guidelines. A descriptive study from Norway. Seizure. 2024;123:92–96. 10.1016/j.seizure.2024.10.00218 39531982

[26] Henning O , Medalen TEM , Nakken KO , Lossius MI . How often do doctors discuss drug withdrawal with their seizure‐free patients with epilepsy? Epilepsy Behav. 2020;108:107095. 10.1016/j.yebeh.2020.107095 32320921

[27] Lee D , Gladwell D , Batty AJ , Brereton N , Tate E . The cost effectiveness of licensed oromucosal midazolam (Buccolam[®]) for the treatment of children experiencing acute epileptic seizures: an approach when trial evidence is limited. Paediatr Drugs. 2013;15(2):151–162. 10.1007/s40272-013-0009-5 23512129

[28] Landmark CJ , Fossmark H , Larsson PG , Rytter E , Johannessen SI . Prescription patterns of antiepileptic drugs in patients with epilepsy in a nation‐wide population. Epilepsy Res. 2011;95(1–2):51–59. 10.1016/j.eplepsyres.2011.02.012 21435840

[29] Baftiu A , Feet SA , Larsson PG , Burns ML , Henning O , Sætre E , et al. Utilisation and polypharmacy aspects of antiepileptic drugs in elderly versus younger patients with epilepsy: a pharmacoepidemiological study of CNS‐active drugs in Norway, 2004–2015. Epilepsy Res. 2018;139:35–42. 10.1016/j.eplepsyres.2017.11.001 29175562

[30] Pisani LR , Nikanorova M , Landmark CJ , Johannessen SI , Pisani F . Specific patient features affect antiepileptic drug therapy decisions: focus on gender, age, and psychiatric comorbidities. Curr Pharm Des. 2017;23(37):5639–5648. 10.2174/1381612823666170926103631 28950817

[31] Midazolam | drugs | BNF | NICE [Internet]. Available from: https://bnf.nice.org.uk/drugs/midazolam/#contra‐indications.

