# Supplementary material for: Rescue epilepsy medication and training: A comparison between midazolam use, guidelines, clinical practice, and possibilities in the UK and Norway
Source: Epilepsia Open. 2025 Oct 6;10(6):1824–34. doi: 10.1002/epi4.70145 (PMC12716287; doi:10.1002/epi4.70145)
Supplement: Supplementary file 1 — Data S1. [file EPI4-10-1824-s008.pdf]

Rescue Epilepsy Medication and Training (REMIT) Survey

Dear Colleague,

We hope this communication finds you and your family/friends well.

We would be grateful for your support in completing our brief survey on seizure rescue therapies and training in the community.

This survey is completed electronically following the link below and will take no more than **15-20 minutes** to compete.

The aim of this survey is to better understand the views on seizure rescue medication prescribing/planning, of healthcare professionals working with people with epilepsy. The survey will uncover common challenges and shared experiences that may then lead to improved patient care.

Context:

The annual incidence of status epilepticus (SE) ranges 10-40/100,000. The aim of seizure rescue medication in the community is to provide a rapidly accessible intervention to reduce the risk of progression to neuronal damage/death and reduce mortality.

In the UK, NICE guidelines detail the emergency pharmacological treatments for seizures in the community. These treatments and their indication may be individualised in an emergency seizure management plan, and are often delivered by non-clinical persons. The formulation and enaction of such emergency management plans has been the subject of significant quality improvement work to enable consistency of care delivery since 2019.

Buccal midazolam is evidenced as the optimal first-line rescue therapy in community settings. However, the maximum dosage, relative and absolute contraindications, use in multi-morbidity, abuse potential and indication for withdrawal remain unclear from a clinical practice perspective.

N.B All questions are optional and majority multiple choice. The survey is designed to keep your replies anonymous. We presume informed consent if you submit the survey. This is a medical student-led project supported by the professionals detailed below. The collated results and analysis of the survey will be fed back either in conferences/ academic papers.

We are very appreciative of your time and contribution to this much-needed work.

If you encounter any issues with the survey or would like to contact the authors please correspond with any of the following individuals:

Ms Audrey McBride - am1531@exeter.ac.uk

Dr Lance Watkins – lance.watkins@plymouth.ac.uk

Professor Rohit Shankar - rohit.shankar@plymouth.ac.uk

Demographics

1. What is your principal clinical job role?

Mark only one oval.

- ⬭ Neurologist - General *Skip to question 2*
- ⬭ Neurologist - Epileptologist *Skip to question 2*
- ⬭ Nurse - Epilepsy Specialist *Skip to question 2*
- ⬭ Nurse - Other *Skip to question 2*
- ⬭ Psychiatrist - Neuropsychiatrist *Skip to question 2*
- ⬭ Psychiatrist - Intellectual Disabilities *Skip to question 2*
- ⬭ Psychiatrist - Other *Skip to question 2*
- ⬭ Paediatrician - General *Skip to question 2*
- ⬭ Paediatrician - Neurologist/ Epileptologist *Skip to question 2*
- ⬭ General Practitioner *Skip to question 2*
- ⬭ Other

Please provide your clinical job role:

Demographics cont.

2. 2. How many years of experience do you have in your epilepsy-related professional role?

Mark only one oval.

- ⬭ 0-3 years
- ⬭ 3-5 years
- ⬭ 5-10 years
- ⬭ 10+ years

3. What proportion of your clinical work is epilepsy-specific?

Mark only one oval.

- ⬭ <25%
- ⬭ 25-50%
- ⬭ 50-75%
- ⬭ >75%

Epilepsy awareness and seizure rescue medication administration training

4. Do you provide training to people with epilepsy, their carers or professionals in epilepsy awareness an[illegible] the administration of seizure rescue therapies?

Mark only one oval.

- ⬭ Yes
- ⬭ No *Skip to question 11*

5. How is training delivered?

Mark only one oval.

- ⬭ Face-to-face
- ⬭ Virtually
- ⬭ Combination face-to-face and virtually

6. How often do you offer training to an individual involved in epilepsy care?

Mark only one oval.

- More than annually
- Annually
- Every 2 years
- Every 3 years or less frequently
- Other: ____________________

Exploring prescribing practices

7. Do you prescribe rescue therapies for people with epilepsy in the community?

Mark only one oval.

- Yes
- No *Skip to question 11*
- Not sure

Exploring prescribing practices cont.

8. Are you a medical or non-medical prescriber?

Mark only one oval.

- Medical prescriber
- Non-medical prescriber

9. Do you request primary care/a GP to prescribe seizure rescue medication for use in the community?

Mark only one oval.

- Yes
- No
- Not sure

10. Do you directly prescribe seizure rescue medication for use in the community?

Mark only one oval.

- Yes
- No
- Not sure

Exploring prescribing practices - emergency management plans cont.

11. When would you suggest/formulate an emergency management plan for individuals experiencing seizures in the community?

(If 'Other' please expand)

Mark only one oval.

- After one episode of status epilepticus
- After an episode of repeated or cluster seizures (typically three or more self-terminating seizures in 2[illegible] horus)
- After a prolonged non-convulsive seizure
- Only after more than one of the above scenarios
- All of the above scenarios
- Other: ______________________

12. How frequently do you <u>routinely</u> plan to review the emergency management plans of people with epilepsy?

Mark only one oval.

- ( ) Six-monthly
- ( ) Annually
- ( ) Less frequently than annually
- ( ) Never
- ( ) N/A

13. What is your opinion on the first-choice rescue therapy for each of the seizure types below, assuming intervention is required? (Only for use in an individual's emergency seizure management plan)

Check all that apply.

	Buccal midazolam	Nasal midazolam	Rectal diazepam	Rectal paraldehyde	Oral clobazam	None	N/A
**Generalised tonic-clonic seizure (prolonged)**	☐	☐	☐	☐	☐	☐	☐
**(Cluster) Generalised tonic-clonic seizures**	☐	☐	☐	☐	☐	☐	☐
**Focal seizures (prolonged)**	☐	☐	☐	☐	☐	☐	☐
**(Cluster) Focal seizures**	☐	☐	☐	☐	☐	☐	☐

Exploring prescribing practices - buccal midazolam

14. What percentage of your caseload is on buccal midazolam?

Mark only one oval.

- <25%
- 25-50%
- 50-75%
- >75%

15. What is the maximum dose of buccal midazolam that you would prescribe for emergency community based seizure termination in a 24 hour period?

Mark only one oval.

- 10mg
- 20mg
- 30mg
- 40mg
- >40mg
- Other: ______________________

16. How likely are you to prescribe buccal midazolam as part of an individual's emergency seizure management plan in people with the following factors?
(1 - Very unlikely
2 - Unlikely
3 - Neutral
4 - Likely
5 - Very likely)

T1DM - Type 1 Diabetes Mellitus

Check all that apply.

	1	2	3	4	5
**Cardiovascular comorbidities**	☐	☐	☐	☐	☐
**Neurological comorbidities**	☐	☐	☐	☐	☐
**History of adverse reactions**	☐	☐	☐	☐	☐
**History of respiratory compromise**	☐	☐	☐	☐	☐
**Person prone to falls**	☐	☐	☐	☐	☐
**Lack of confidence in buccal midazolam administrator**	☐	☐	☐	☐	☐
**Risk of drug abuse/dependence**	☐	☐	☐	☐	☐
**Alcohol related seizures**	☐	☐	☐	☐	☐
**Known issues with compliance**	☐	☐	☐	☐	☐
**Social issues (lack of carer support)**	☐	☐	☐	☐	☐
**Pregnant women**	☐	☐	☐	☐	☐
**People with T1DM**	☐	☐	☐	☐	☐
**People with Intellectual Disabilities**	☐	☐	☐	☐	☐

17. Have you ever been concerned that buccal midazolam is being used inappropriately?

Mark only one oval.

- Yes *Skip to question 18*
- No
- Not sure *Skip to question 18*

Buccal midazolam question follow up

18. Please share your experience regarding the inappropriate use of buccal midazolam:

____

Exploring prescribing practices - deprescribing buccal midazolam

19. After what period of non-use do you think that buccal midazolam should be withdrawn from an individual's emergency management plan?

Check all that apply.

- [ ] 1 year
- [ ] 2 years
- [ ] 3 years
- [ ] Never
- [ ] Other: ________________________________

20. Please give your rationale for deprescribing after your selected time period:

This content is neither created nor endorsed by Google.

Google Forms
