# Supplementary material for: Rescue epilepsy medication and training: A comparison between midazolam use, guidelines, clinical practice, and possibilities in the UK and Norway
Source: Epilepsia Open. 2025 Oct 6;10(6):1824–34. doi: 10.1002/epi4.70145 (PMC12716287; doi:10.1002/epi4.70145)
Supplement: Supplementary file 2 — Table S1. [file EPI4-10-1824-s006.docx]

Table S1. Checklist for Reporting Results of Internet E-Surveys (CHERRIES)

|  | **Checklist for Reporting Results of Internet E-Surveys (CHERRIES)** | |
| --- | --- | --- |
| ***Item Category*** | ***Checklist Item*** | ***Explanation*** |
| **Design** |  |  |
|  | Describe survey design | “It was designed for completion by any registered healthcare professionals working with patients with epilepsy”   - open, voluntarily completed on visiting the site and unincentivized. |
| **IRB (Institutional Review Board) approval and informed consent process** |  |  |
| N/A no ethics required | IRB approval | N/A |
|  | Informed consent | Informed consent was gained from participants via the first screen’s letter of introduction (Appendix C). Contact details were provided for participants with queries. |
| N/A - no storage | Data protection | Demographic data including job role, responsibilities, and length of experience with epilepsy was collected from participants.  Anonymised data stored offline (PB). |
| **Development and pre-testing** |  |  |
|  | Development and testing | “The survey items were developed iteratively, through informal interviews of specialists in epilepsy management and reviews of key documents regulating rescue therapies in the UK.^6,7,10^ An initial draft (AM) was collaboratively revised in five rounds of revision (AM, RS, LW) between July and September 2023. The survey was then hosted online and completed by key stakeholders at the Epilepsy Specialist Nurse’s Association (ESNA) to test its content and usability, and finalised after their feedback.” |
| **Recruitment process and description of the sample having access to the questionnaire** |  |  |
| Open | Open survey versus closed survey | The survey was published via the Google Forms online platform. It was open, voluntarily completed on visiting the site and unincentivized. |
|  | Contact mode | “The survey was distributed through regular newsletter emails to members of ESNA and the ILAE and concurrently through snowball sampling of intellectual disability psychiatrists and neurologists. Promotional materials can be found as a supplementary item (Appendix B” |
|  | Advertising the survey | “The survey was distributed through regular newsletter emails to members of ESNA and the ILAE and concurrently through snowball sampling of intellectual disability psychiatrists and neurologists. Promotional materials can be found as a supplementary item (Appendix B” |
| **Survey administration** |  |  |
| Google forms | Web/E-mail | The survey was published via the Google Forms online platform. |
| N/A | Context | A web-based search engine extension app designed to run surveys. |
| Voluntary | Mandatory/voluntary | Voluntary |
|  | Incentives | Unincentivized |
|  | Time/Date | “The survey was open for 12 weeks, between 13/10/2023 and 29/12/2023.” |
|  | Randomization of items or questionnaires | Question items were not randomised but question answers were randomised unless they followed a numerical sequence. |
|  | Adaptive questioning | “The survey comprised 21 question items in total, 14 of which were presented to all participants and seven of which were adaptive (Appendix C)." |
|  | Number of Items | Items were spread across eight or nine screens and included 18 multiple choice and three free-text responses. Full survey layout would have varied by participant due to adaptive questioning. The full survey can be found in Appendix C. |
|  | Number of screens (pages) | Items were spread across eight or nine screens and included 18 multiple choice and three free-text responses. |
|  | Completeness check | The completeness rate can be found in supplementary materials (Appendix D). All questionnaires were analysed, regardless of completeness. |
|  | Review step | Participants were able to review and change their answers with a back button, but not after submission. |
| **Response rates** |  |  |
|  | Unique site visitor | The uniqueness of site viewers and participants was not evaluated. |
|  | View rate (Ratio of unique survey visitors/unique site visitors) | The view rate was not measured. |
|  | Participation rate (Ratio of unique visitors who agreed to participate/unique first survey page visitors) | The participation rate was not measured. |
|  | Completion rate (Ratio of users who finished the survey/users who agreed to participate) | The completion rate was 98%. |
| **Preventing multiple entries from the same individual** |  |  |
|  | Cookies used | Not used. |
| Not applicable, not used | IP check | Not used. |
|  | Log file analysis | Not used. |
|  | Registration | Not used. |
| **Analysis** |  |  |
|  | Handling of incomplete questionnaires | All questionnaires were analysed, regardless of completeness. |
|  | Questionnaires submitted with an atypical timestamp | Not used. |
|  | Statistical correction | Not used. |
