# Supplementary material for: Rescue epilepsy medication and training: A comparison between midazolam use, guidelines, clinical practice, and possibilities in the UK and Norway
Source: Epilepsia Open. 2025 Oct 6;10(6):1824–34. doi: 10.1002/epi4.70145 (PMC12716287; doi:10.1002/epi4.70145)
Supplement: Supplementary file 3 — Table S2. [file EPI4-10-1824-s005.docx]

Table S2. Checklist for Reporting Results of Internet E-Surveys (CHERRIES)

|  | **Checklist for Reporting Results of Internet E-Surveys (CHERRIES)** | |
| --- | --- | --- |
| ***Item Category*** | ***Checklist Item*** | ***Explanation*** |
| **Design** |  |  |
|  | Describe survey design | “It was designed for completion by any registered healthcare professionals working with patients with epilepsy”   - open, voluntarily completed on visiting the site and unincentivized. |
| **IRB (Institutional Review Board) approval and informed consent process** |  |  |
| Approved | IRB approval | Approved by IRB: reference 24/01100 |
|  | Informed consent | Informed consent was gained from participants via the first screen’s letter of introduction. Contact details were provided for participants with queries. |
| N/A - no storage | Data protection | Demographic data including job role, responsibilities, and length of experience with epilepsy was collected from participants.  Anonymised data stored offline |
| **Development and pre-testing** |  |  |
|  | Development and testing | “The survey items were developed iteratively, through informal interviews of specialists in epilepsy management and reviews of key documents regulating rescue therapies in the UK. The survey was translated to Norwegian and reviewed by Epileptologists from the national centre of epilepsy in Norway and some questions were added in the Norwegian version of the survey. |
| **Recruitment process and description of the sample having access to the questionnaire** |  |  |
| Open | Open survey versus closed survey | The survey was published via Questback. It was open, voluntarily completed on visiting the site and unincentivized. |
|  | Contact mode Advertising the survey | A link to the survey was distributed through collaboration with key stakeholder organisations like the Norwegian chapter of the International League Against Epilepsy (ILAE) (NES), the national network for epilepsy nurses, the national interdisciplinary epilepsy network (Epilepsinet) and through local and national communities. |
| **Survey administration** |  |  |
| Questback | Web/E-mail | A link to the survey was distributed |
| N/A | Context | A web-based survey app. |
| Voluntary | Mandatory/voluntary | Voluntary |
|  | Incentives | Unincentivized |
|  | Time/Date | The survey was open between 01/06/2024 and 05/09/2024 |
|  | Randomization of items or questionnaires | Question items were not randomised. |
|  | Adaptive questioning | The survey comprised 20 question items in total, 16 of which were presented to all participants and four of which were adaptive |
|  | Number of Items | Items included 4 single choice 14 multiple choice and two free-text responses. |
|  | Number of screens (pages) | Items were spread across four screens. |
|  | Completeness check | The completeness rate can be found in supplementary. All questionnaires were analysed, regardless of completeness. |
|  | Review step | Participants were able to review and change their answers with a back button, but not after submission. |
| **Response rates** |  |  |
|  | Unique site visitor | The uniqueness of site viewers and participants was not evaluated. |
|  | View rate (Ratio of unique survey visitors/unique site visitors) | The view rate was not measured. |
|  | Participation rate (Ratio of unique visitors who agreed to participate/unique first survey page visitors) | The participation rate was not measured. |
|  | Completion rate (Ratio of users who finished the survey/users who agreed to participate) | The completion rate was 98%. |
| **Preventing multiple entries from the same individual** |  |  |
|  | Cookies used | Not used. |
| Not applicable, not used | IP check | Not used. |
|  | Log file analysis | Not used. |
|  | Registration | Not used. |
| **Analysis** |  |  |
|  | Handling of incomplete questionnaires | All questionnaires were analysed, regardless of completeness. |
|  | Questionnaires submitted with an atypical timestamp | Not used. |
|  | Statistical correction | Not used. |
