# Supplementary material for: Rescue epilepsy medication and training: A comparison between midazolam use, guidelines, clinical practice, and possibilities in the UK and Norway
Source: Epilepsia Open. 2025 Oct 6;10(6):1824–34. doi: 10.1002/epi4.70145 (PMC12716287; doi:10.1002/epi4.70145)
Supplement: Supplementary file 4 — Table S3. [file EPI4-10-1824-s003.docx]

*Table S3: Study participant demographics - UK*

| Variable | N | Category | Number (f) | Number (%) |
| --- | --- | --- | --- | --- |
|  |  |  |  |  |
| Clinical role | 86 | Neurologist – epileptologist | 5 | (6%) |
|  |  | Neurologist – general | 1 | (1%) |
|  |  | Paediatrician – general | 2 | (2%) |
|  |  | Paediatrician – neurologist | 3 | (3%) |
|  |  | Psychiatrist | 10 | (12%) |
|  |  | Nurse – epilepsy specialist | 52 | (60%) |
|  |  | Nurse – other | 12 | (14%) |
|  |  | Other | 1 | (1%) |
|  |  |  |  |  |
| Job category | 86 | Medic | 21 | (24%) |
| (combined) |  | Nurse | 64 | (74%) |
|  |  | Other | 1 | (1%) |
|  |  |  |  |  |
| Experience in | 86 | 0 – 3 years | 16 | (19%) |
| epilepsy-related role |  | 3 – 5 years | 17 | (20%) |
|  |  | 5 – 10 years | 11 | (13%) |
|  |  | 10+ years | 42 | (49%) |
|  |  |  |  |  |
| Epilepsy-specific | 86 | < 25% | 7 | (8%) |
| work |  | 25% – 50% | 15 | (17%) |
|  |  | 50% - 75% | 11 | (13%) |
|  |  | > 75% | 53 | (62%) |
|  |  |  |  |  |
