# Supplementary material for: Rescue epilepsy medication and training: A comparison between midazolam use, guidelines, clinical practice, and possibilities in the UK and Norway
Source: Epilepsia Open. 2025 Oct 6;10(6):1824–34. doi: 10.1002/epi4.70145 (PMC12716287; doi:10.1002/epi4.70145)
Supplement: Supplementary file 5 — Table S4. [file EPI4-10-1824-s001.docx]

*Table S4: Study participant demographics – Norway*

| Variable | N | Category | Number (%) |
| --- | --- | --- | --- |
|  |  |  |  |
| Clinical role | 53 | Neurologist – epileptologist | 13 (25%) |
|  |  | Neurologist – general | 13 (25%) |
|  |  | Neuropaediatrician | 4 (8%) |
|  |  | Nurse – epilepsy specialist | 9 (17%) |
|  |  | Nurse – other | 12 (23%) |
|  |  | Clinical nutritionist | 1 (2%) |
|  |  | Special education teacher | 1 (2%) |
|  |  |  |  |
| Job category | 53 | Medic | 30 (57%) |
| (combined) |  | Nurse | 21 (40%) |
|  |  | Other | 2 (4%) |
|  |  |  |  |
| Experience in | 53 | 0 – 3 years | 1 (2%) |
| epilepsy-related role |  | 3 – 5 years | 7 (13%) |
|  |  | 5 – 10 years | 7 (13%) |
|  |  | 10+ years | 38 (72%) |
|  |  |  |  |
| Epilepsy-specific | 53 | < 25% | 8 (15%) |
| work |  | 25% – 50% | 6 (11%) |
|  |  | 50% - 75% | 9 (17%) |
|  |  | > 75% | 30 (57%) |
|  |  |  |  |
