# Supplementary material for: Rescue epilepsy medication and training: A comparison between midazolam use, guidelines, clinical practice, and possibilities in the UK and Norway
Source: Epilepsia Open. 2025 Oct 6;10(6):1824–34. doi: 10.1002/epi4.70145 (PMC12716287; doi:10.1002/epi4.70145)
Supplement: Supplementary file 6 — Table S5. [file EPI4-10-1824-s007.docx]

*Table S5: Initiation and review of emergency management plans UK vs Norway (all respondents)*

| Variable | Category | UK | | Norway | | P- |
| --- | --- | --- | --- | --- | --- | --- |
|  |  | N | Number (%) | N | Number (%) | value |
|  |  |  |  |  |  |  |
| Plan in all situations: | No | 85 | 27 (32%) | 52 | 19 (37%) | 0.57 |
| one episode of SE, an episode of cluster seizures and a prolonged non-convulsive seizure | Yes |  | 58 (68%) |  | 33 (63%) |  |
|  |  |  |  |  |  |  |
| Frequency of plan review | 6-montly | 83 | 8 (10%) | 37 | 5 (14%) | **0.006** |
|  | Annually |  | 64 (77%) |  | 15 (41%) |  |
|  | < annually |  | 10 (12%) |  | 16 (43%) |  |
|  | Never |  | 1 (1%) |  | 1 (3%) |  |
|  |  |  |  |  |  |  |
