# Supplementary material for: Rescue epilepsy medication and training: A comparison between midazolam use, guidelines, clinical practice, and possibilities in the UK and Norway
Source: Epilepsia Open. 2025 Oct 6;10(6):1824–34. doi: 10.1002/epi4.70145 (PMC12716287; doi:10.1002/epi4.70145)
Supplement: Supplementary file 7 — Figure S1. [file EPI4-10-1824-s002.docx]

*Figure S1: Buccal midazolam items, UK vs Norway (physicians only)*


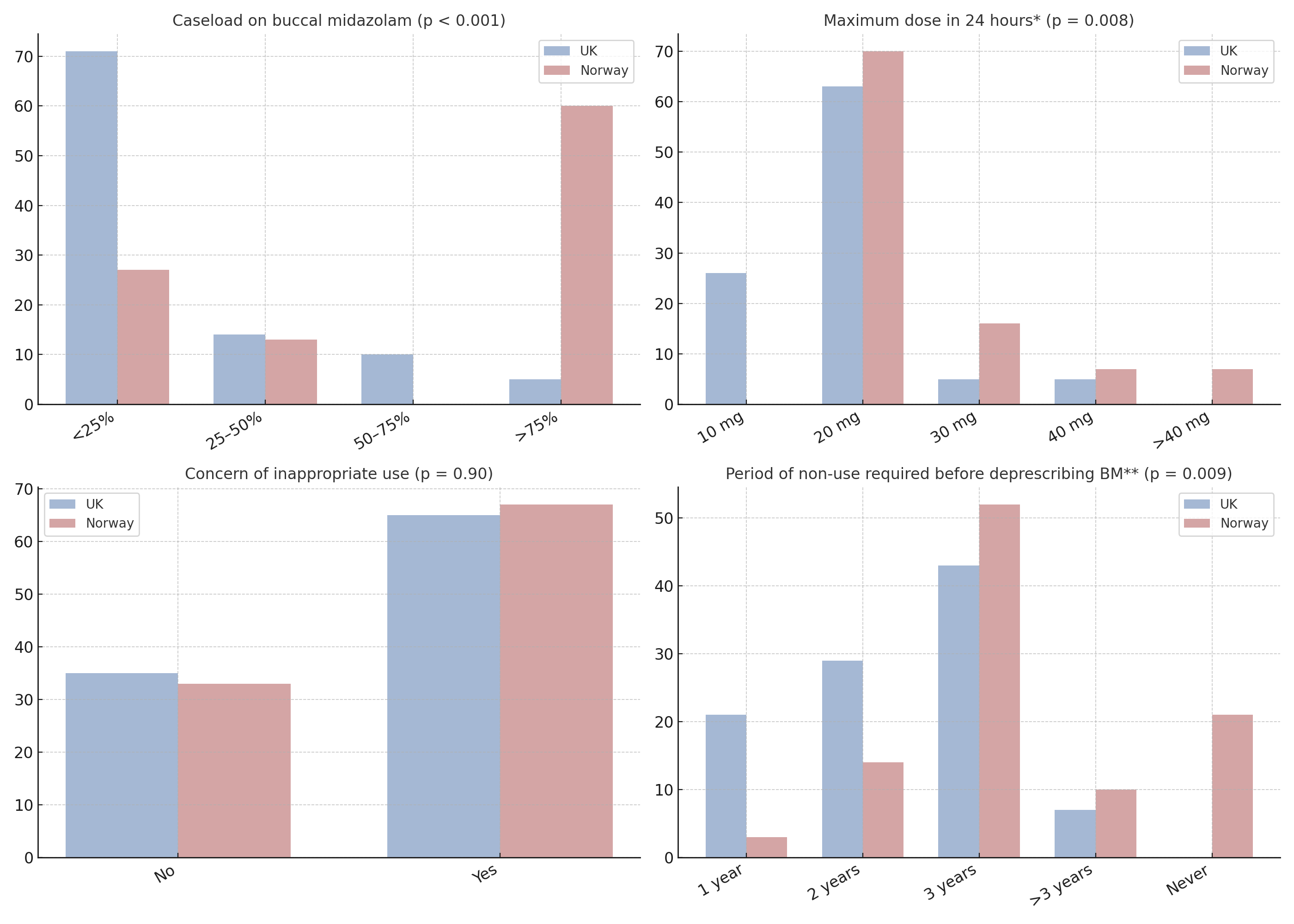


(*) Omitting 2 physicians from UK with ‘other’ response from statistical comparison due to unknown exact value for these respondents

(**) Omitting data from 7 physicians from UK and 1 physician from Norway who gave ‘other’ responses without a specific time for withdraw

Y-axis = percentage respondents
