# Supplementary material for: Rescue epilepsy medication and training: A comparison between midazolam use, guidelines, clinical practice, and possibilities in the UK and Norway
Source: Epilepsia Open. 2025 Oct 6;10(6):1824–34. doi: 10.1002/epi4.70145 (PMC12716287; doi:10.1002/epi4.70145)

*Figure S2: Likelihood of prescribing buccal midazolam in different scenario, UK vs Norway (physicians only)*
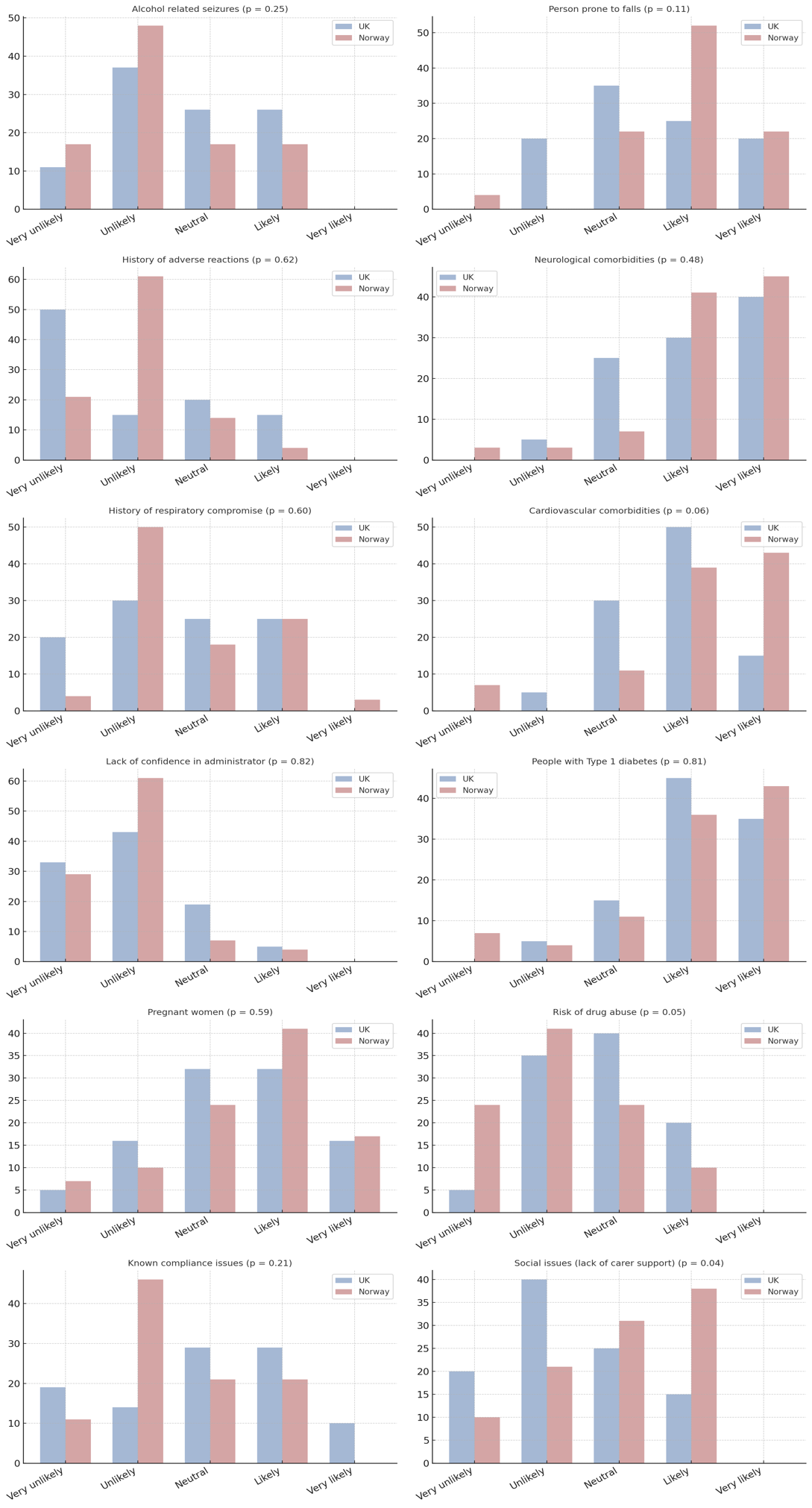

Supplement: Supplementary file 8 — Figure S2. [file EPI4-10-1824-s004.docx]
